# Family Planning Policy Environment in the Democratic Republic of the Congo: Levers of Positive Change and Prospects for Sustainability

**DOI:** 10.9745/GHSP-D-14-00244

**Published:** 2015-06-12

**Authors:** Thibaut Mukaba, Arsene Binanga, Sarah Fohl, Jane T Bertrand

**Affiliations:** ^a^​ United States Agency for International Development/Democratic Republic of the Congo, Kinshasa, DRC; ^b^​ Tulane International LLC, Kinshasa, DRC; ^c^​ Tulane University School of Public Health and Tropical Medicine, Department of Global Health Systems and Development, New Orleans, LA, USA

## Abstract

Building on expressed support from the Prime Minister to the Ministries of Health and Planning, the country’s new family planning commitment grew out of: (1) recognition of the impact of family planning on maternal mortality and economic development; (2) knowledge sharing of best practices from other African countries; (3) participatory development of a national strategic plan; (4) strong collaboration between stakeholders; (5) effective advocacy by champions including country and international experts; and (6) increased donor support. The question becomes: Will the favorable policy environment translate into effective local programming?

## INTRODUCTION

The Democratic Republic of the Congo (DRC)—with a population estimated at 67.5 million^1^—is the third most populous country in sub-Saharan Africa and the most populous francophone country. Since its independence from Belgium in 1960, the country has had a tumultuous history.

During the dictatorship of President Mobutu Sese Seko (1965–1997), when the country was named Zaire, the repressive government provided periods of relative stability but at great cost to the country and to its citizens. In 1991, Mobutu lost control over the population; political turmoil and social unrest, known as the *pillage* (plundering), created a highly volatile environment that caused foreign investors and international donor agencies to withdraw completely or reduce support to the country, plunging the country further into poverty. The First Congo War (1996–1997) resulted in Mobutu being overthrown by the Rwandan-backed rebel leader Laurent-Desiré Kabila.^2^

The Second Congo War (that involved 9 neighboring countries) occurred primarily in the eastern part of the country between 1998 and 2002, resulting in further economic devastation. By the early 2000s, life in the capital city of Kinshasa and some other provinces began returning to normalcy, although the threat of war continued to simmer in the eastern provinces bordering Rwanda and Uganda.^3^ Because of the ravages of war, political uncertainty, external exploitation, inadequate investment in human capital, and widespread poverty, the DRC—one of the richest countries in the world in minerals—ties for last place among 187 nations on the Human Development Index.^4^

The DRC is typical of African countries that are just beginning the demographic transition, with a total fertility rate of 6.6 as of 2013,^5^ a doubling in population size every 23 years, and a very young population (46% are under the age of 15). Almost three-quarters (71.3%) of the population live below the poverty line, and 45.9% live in severe poverty.^5^ With its high maternal mortality rate of 846 per 100,000 live births^5^ and large population base, the DRC is among the 5 countries that contribute the greatest number of maternal deaths to the global total.^6^

Although the country established a national family planning program (*Projet National des Naissances Desirables*, or PSND) in the early 1980s, it became inactive in the early 1990s in the wake of the *pillage*. In the mid2000s, external donors and technical organizations tentatively began to resume operations in the DRC. With a myriad of challenges facing the government from every sector, family planning was low on the priority list; the government had more important problems to address. Although the government established the National Program of Reproductive Health (*Programme National de Santé de la Reproduction*, or PNSR) in 2001 to address maternal mortality, family planning, and related issues, it drew little attention or support from higher levels of government. While maternal mortality did register as a national priority, family planning did not during the first decade of the millennium. The Growth and Poverty Reduction Strategy failed to mention family planning, although it did touch on leprosy, onchocercosis, and tuberculosis^7^; family planning was mentioned in the second edition released in 2010. For decades, there was little evidence of government interest in family planning.

Some have questioned if the Roman Catholic Church has been an obstacle to family planning in the DRC. The Ministry of Health estimates that 40% of health facilities in the DRC are managed by the Church.^8^ In some cases, these facilities refuse to offer modern contraceptive methods; in others, they turn a blind eye. To the extent that the Roman Catholic Church in the DRC has attempted to exercise its influence in terms of family planning, it has been to promote natural methods over “artificial contraception.” In contrast to selected countries in Latin America and to the Philippines (where the Church has tried to block government or NGO family planning programs), it does not constitute a source of organized opposition to family planning programming in the DRC.

Beginning in 2012, the DRC government took a number of actions that demonstrated its commitment to family planning, which has taken the international family planning community and many local observers by surprise. The purpose of this article is to document the evolution of the family planning policy environment in the DRC, analyze events that led to positive change, and identify factors that could influence the durability of this change.

## PREVIOUS ATTEMPTS TO HEIGHTEN THE PROFILE OF FAMILY PLANNING

It is useful to begin the analysis of the family planning policy environment in the DRC in 2004, as the country was beginning to return to normalcy after the *pillage* and subsequent political turmoil. Two key conferences to reposition family planning occurred in 2004 and 2009 that typified the lack of high-level support for family planning and inability of the PNSR to mobilize and sustain political support for family planning.

The first *Conférence pour le Repositionnement de la Planification Familiale* (Conference on Repositioning Family Planning), held in 2004, was funded by the United States Agency for International Development (USAID) and executed by the PNSR with technical assistance from the Advance Africa Project.^9^ Given the country’s ongoing struggle to return to normalcy, perhaps it was premature to expect tangible results to come from this gathering.

The second National Conference on Repositioning Family Planning in the Democratic Republic of Congo took place in 2009, again with support from USAID in addition to the United Nations Population Fund (UNFPA).^10^ This costly event, held at the Ministry of Foreign Affairs conference room, took place under the auspices of the First Lady of the DRC. At a time when programs in other health sectors (e.g., HIV, malaria, vaccination) were receiving major funding and government attention, the second conference on repositioning family planning was designed to heighten visibility for family planning through the high-level endorsement by the First Lady and to promote public dialogue around its importance. One tangible benefit of the conference was that it called for establishing a multisectoral committee of family planning stakeholders (*Comité Technique Multisectoriel Permanent*, CTMP), which proved to be very useful. However, overall, the conference produced no immediate results in terms of political support or programmatic action. In retrospect, a major investment of time, effort, and resources went into planning and implementing this event, with insufficient attention to substantive follow-up. For example, the First Lady was not engaged to play a role in subsequent events, although she is still cited as a champion of family planning.

Few tangible benefits resulted from holding national family planning conferences because insufficient attention was paid to substantive follow-up.

In 2007, between the two national family planning conferences, USAID established a Family Planning Partners’ Group, including its own implementing partners and the PNSR. This group improved coordination within the USAID program; however, the PNSR lacked the capacity to organize coordination at the national level. In 2008, the PNSR created the Family Planning Task Force, which was joined by members from USAID's Family Planning Partners' Group. When the CTMP was established following the 2009 conference, it included many of the same players as the Family Planning Task Force, at which point the task force ceased to exist.

## FACTORS CONTRIBUTING TO THE EVOLVING SUPPORT FOR FAMILY PLANNING

Since 2012, the DRC government has boldly endorsed family planning in a number of actions (Box 1). In particular, the letters in late 2013 from the Prime Minister’s office to the Ministry of Health and Ministry of Planning reflected support from the highest levels of government, which triggered several subsequent public commitments to family planning. How did the change from near virtual neglect to explicit, strong support of family planning by the DRC government occur in a period of approximately 2 years? This question does not lend itself to statistical analysis, but a review of relevant events points to the following factors.

2012 marks a turning point in the DRC’s support for family planning—from near virtual neglect only 2 years prior.

**BOX 1. Evidence of DRC Government Commitment to Family Planning Since 2012**

**2012:**

- **June.** The DRC government included family planning as 1 of 6 elements in its framework to accelerate achievement of Millennium Development Goal No. 4 (reduce child mortality) and No. 5 (improve maternal health).
- **August.** The Cadre Permanent de Concertation des Femmes Congolaises (CAFCO, or the Permanent Consultative Framework of Congolese Women) drafted a law favorable to reproductive health/family planning.
- October 15. PNSR, with support from UNFPA and other partners in Kinshasa, launched a family planning campaign in Masina municipality (consisting of intensified mass communication and free distribution of contraceptives).
- **December 13.** CAFCO submitted the draft reproductive health/family planning law to the National Assembly for consideration.

**2013:**

- **February.** DRC government allocated US$994,000 from the national budget toward the procurement of contraceptives, with subsequent allocations conditional on receipt of initial order.
- **July 29.** The Prime Minister sent a letter to the Minister of Health, with a copy to the Minister of Planning, to prioritize family planning, citing the negative socioeconomic impact of population growth if the issue of high fertility was not addressed appropriately.
- **August 21.** The Minister of Health sent a letter of response to the Prime Minister regarding prioritizing family planning in the 2014 budget and taking explicit steps toward strengthening work in family planning. The Minister of Budget also sent a letter of response to the Prime Minister stating that the Ministry of Budget would allocate funding for family planning in the 2014 budget.
- **November 15.** The DRC Government presented the Declaration of Commitment to Family Planning at the Third International Conference on Family Planning in Addis Ababa.
- **November 28.** The Prime Minister sent a second letter to the Minister of Health declaring the importance of finalizing the National Strategic Plan for Family Planning, formalizing the Permanent Multisectoral Technical Committee (CTMP), and developing performance contracts for PNSR staff.
- **Exact date unknown.** National Assembly selected the draft reproductive health/family planning law for discussion in the March 2014 session of the National Assembly.

**2014:**

- **January 10.** The DRC government officially approved the National Strategic Plan for Family Planning in the DRC: 2014–2020.
- **February 13.** The Minister of Health officially launched the National Strategic Plan for Family Planning.
- **March-November.** The government held additional deliberations on the proposition of the reproductive health/family planning law.
  - **March.** The National Assembly sent the proposition to the Government for Avis Technique (technical opinion).
  - **March.** The Government forwarded the proposition to the Minister of Health to supply the Avis Technique.
  - **April 4.** The Minister of Health provided the Avis Technique to the Government.
  - **June 12.** Two members of the National Assembly-who are also members of CAFCO-presented the draft reproductive health/family planning law to the National Assembly for deliberation.
  - **August 28.** The National Assembly sent the proposition to the Supreme Court to determine whether the National Assembly is authorized to legislate on the proposition.
  - **November.** The Supreme Court of Justice sent the proposition to the Parquet Général de la Republique (National General Prosecutor's office) for consultation.
  - **November.** The Parquet Général de la Republique returned the proposition to the Supreme Court to pronounce whether the National Assembly should legislate on this topic.
- **December 3–5, 2014.** The Office of the Prime Minister sponsored the Third National Conference on Repositioning Family Planning in the DRC, which was attended by more than 400 participants, including provincial ministers from the 11 provinces.
  - The Minister of Health presided over the opening and closing ceremony as well as a roundtable.
  - The Minister of Planning presided over both a plenary session on Day 1 and a roundtable.
  - The DRC government and donors attended the roundtable on December 5, at which the DRC government doubled its family planning commitment in 2015 to US$2.5 million and representatives from multilateral/bilateral agencies and foundations declared commitments to family planning in the coming years.

### Heightened Awareness of Family Planning as a Means to Reduce Maternal Mortality

In June 2012, the Minister of Health and other technical advisors attended the first meeting on the Child Survival Call to Action: A Promise Renewed,^11^ which included participants from countries with some of the highest maternal mortality rates in the world, as well as those from countries currently on track to achieving Millennium Development Goal (MDG) No. 4 (reduce child mortality) and No. 5 (improve maternal health). One aim of the meeting was to encourage the participating countries to be more accountable for these high levels of maternal mortality and to discuss strategies for taking action. Each country was asked to develop a plan to address the question of what it was doing to reduce infant and child mortality. These same parties, as well as the director of the Health Office of USAID/DRC, participated in a follow-up meeting in Kinshasa in late 2012.

Regional family planning conferences improved DRC stakeholder knowledge of best practices from other African countries.

The DRC government, with support from the United Nations Children’s Fund (UNICEF), USAID, the World Health Organization (WHO), and others, responded by developing the “Plan for Accelerating Achievement of the MDGs,” in which family planning was 1 of 6 actions to achieve MDG 4 and 5. This plan provided one of the first signs that the DRC government intended to pay more than lip service to family planning.

### Strengthened Ties Between Partners

Although the PNSR is a government entity, historically its dealings with higher levels of government were limited and perfunctory. However, beginning in 2012 ties strengthened between the PNSR and the government bodies that it represents (10ème Direction [D10], Ministry of Health). Also, an expert within the *Direction d’Etudes et Planification* (DEP) (Studies and Planning Directorate) came forward as a champion for family planning. Although responsible for multiple areas of health, he strategically used his time and influence to participate in key meetings, intercede on behalf of the PNSR, and liaise with higher-level government officials on issues related to family planning. He also recognized the role that external partners could play in advancing the family planning agenda within the country and embraced their participation, although doing so on his own terms.

### Increased Awareness About Best Practices From Other African Countries

Between 2008 and 2012, USAID invited PNSR, the *Programme National de Santé de l'Adolescent* (PNSA) (National Adolescent Health Program), and key implementing partners to participate in successive regional family planning meetings held in Kenya, Rwanda, and Tanzania, that convened up to 14 African countries. Participants at the regional meetings discussed strategies for meeting the family planning demand to achieve the MDGs, effective community approaches to family planning, and using mobile technology to improve family planning and health services. In addition, the same DRC organizations attended the International Conference on Family Planning in Kampala in 2009 and in Dakar in 2011. Attending these conferences not only improved DRC stakeholders’ knowledge of successful family planning models from across the continent but also reinforced ties among family planning activists in the DRC and facilitated collaboration within coordination bodies that were created later.

Awareness of family planning’s impact on reducing maternal mortality propelled the DRC government to take action.

### Greater Cohesiveness and Teamwork Among Technical Partners

Prior to 2010, some 10 organizations (funded by USAID, UNFPA, the World Bank, the Department for International Development [DFID], the International Planned Parenthood Federation [IPPF], and others) implemented family planning service delivery in Kinshasa, albeit in a relatively isolated manner. Others operated in the 10 provinces outside Kinshasa. Each organization diligently pursued individual projects or institutional objectives, but none (including the PNSR) was addressing the larger questions: How can we increase the availability of contraceptive methods? Can we increase contraceptive use? The PNSR was unable to operate effectively in its convening capacity, given insufficient human and financial resources.

Two coordinating mechanisms based in Kinshasa—the CTMP and the Kinshasa Family Planning Coalition—helped to bring greater cohesiveness among these different partners and greater collaboration with the PNSR in the capital city of Kinshasa. The CTMP—mandated by the 2009 Conference on Repositioning Family Planning—gained a new level of functionality as it planned a conference in Kinshasa in June 2012. The conference, entitled “Advocacy for the Financing of Family Planning in the DRC,” aimed at increasing awareness of the work of different partners in promoting family planning.^9^ In late 2013, the CTMP became the main liaison between the organizers of the 2013 International Conference on Family Planning in Addis Ababa and the delegation from the DRC, which numbered more than 40 participants. In addition, the CTMP served as a key intermediary in organizing the presentation of the DRC government’s declaration of commitment at the Addis meeting. Once in Addis, the CTMP arranged meetings with the DRC delegation (including government and NGO representatives) and foundation representatives, as well as a press conference with international correspondents. In February 2015, the Office of the Prime Minister gave official status to the CTMP, further strengthening its mandate as a coordinating body.

The second group that has brought greater cohesiveness among the organizations implementing family planning service delivery is the Kinshasa Family Planning Coalition. Since its creation in December 2012, this group of 10 service delivery organizations and 4 key donors has met quarterly. In 2013, the group aimed to increase the percentage of health structures offering “3-star” quality services from 44% (determined by a 2012 baseline survey) to 80%. Results from a follow-up survey showed that by the end of 2013, the number of health facilities reporting that they provided family planning services increased from 184 to 395. The percentage of health facilities with a 3-star quality rating increased from 44% to 63% among the total number of sites surveyed in 2013.^12^ Although short of the 80% aspirational goal, this significant increase provided evidence that local organizations could effectively increase access to and quality of family planning services in Kinshasa.

Donor-funded implementing partners have had a major role in advancing the family planning agenda to date. For example, external donors bear 85% of the costs of contraceptive procurement in the DRC, while households shoulder 15% of the costs and the DRC government less than 1%.^13^ While commitment of donors is commendable, eventual success of the national family planning program must involve solid collaboration between the government and the private sector, including international and local NGOs and faith-based organizations.

The strength of the ongoing collaboration among multiple donors and partner organizations was evident in the organization and execution of the Third National Conference on Repositioning Family Planning in the DRC, held in Kinshasa from December 3–5, 2014, and attended by more than 400 participants. The event included 4 plenary sessions, 12 parallel technical sessions, 8 mini-workshops, and 2 consultative sessions with more than 40 officials representing the 11 provinces of the DRC. Sponsored by the Office of the Prime Minister and opened by the Minister of Planning, this event had strong government representation. It culminated with a roundtable of donors and ministry officials. The Ministers of Planning and Health jointly presided over this event, in which the DRC government doubled its commitment to the procurement of contraceptives from the previous year to $2.5 million for 2015.

### Development of a National Strategic Family Planning Plan

In 2012, the CTMP took on the task of developing the National Strategic Plan for Family Planning, soliciting and obtaining financial assistance to support the process from various implementing partners, thus drawing in multiple parties. The President of the CTMP led the highly participatory process, consisting of 4 workshops between December 2012 and October 2013 and involving more than 200 participants from the provinces and from different ministries. In the fourth and final workshop, the Provincial Ministers of Health and Provincial Medical Directors (*Médecins Inspecteurs*) from the 11 provinces of the DRC were invited to Kinshasa to discuss the key components of the plan.

Using a participatory process to develop the national strategic family planning plan helped create buy-in.

Because the large majority of family planning stakeholders, especially those based in Kinshasa, had participated in one or more of the workshops, the final strategic plan had considerable buy-in throughout the family planning community. The final draft was ready just in time for the Addis Ababa International Conference on Family Planning, which gave added weight and credibility to the government’s declaration of commitment to family planning. The national strategic plan outlined objectives and sub-objectives, along with concrete activities to achieve them at specified points in time (in other words, a roadmap for increasing contraceptive prevalence in the DRC). The Minister of Health participated in the process at regular intervals and presided over the high-profile launch of the strategic plan on February 13, 2014, where he gave a very impassioned public endorsement of it.

### Informed Advocacy From International Experts

The presence of international experts with a keen interest in the DRC combined with years of experience in other African countries allowed them to effectively advocate increased support for family planning from different vantage points. Three family planning champions, in particular, contributed to the momentum in the DRC that dates back to 2012 (Box 2). These individuals ensured that family planning was on the public health agenda at a time when there was very little public discourse about family planning, made visible the economic benefits of lower fertility rates, and encouraged partners on the ground that improving access to and use of family planning in the country was in fact possible.

**BOX 2. Champions Contributing to the Family Planning Momentum in the DRC**

Many individuals, both local and international, have contributed to the family planning momentum in the DRC. Here, we highlight the contributions of 3 international family planning experts in particular.

Dr. Richard Dackam-Ngatchou served as UNFPA Representative for the DRC from 2010–2012. Within UNFPA, he gave heightened priority to family planning activity. Moreover, he lost no opportunity to advocate family planning in private meetings with government officials and in public fora with large audiences. At a time when there was too little public discourse on family planning, Dr. Dackam-Ngatchou ensured this topic remained part of the public health agenda.

Dr. Jean-Pierre Guengant, demographer and economist from the Université Paris 1 Panth´on-Sorbonne, has performed analyses involving population projections, socioeconomic consequences, and the demographic dividend in countries throughout francophone Africa (including for all 9 countries participating in the Ouagadougou Partnership).14 Although he visited the DRC several times in the early 2000s, he returned in 2012 to continue this work in a more sustained manner, meeting periodically with different government officials and presenting his findings to selected audiences. In addition, he directly contributed to the costing section of the Strategic Plan. His research, published in 2014 under the title Bénéficier du Dividende Démographique? Replacer la population au centre des trajectoires de développement de la République Démocratique du Congo,15 provided the evidence base for public dialogue on the demographic dividend. It was the lead theme at the December 2014 Third National Conference on Repositioning Family Planning in the DRC, which gave a highly visible platform to the linkage between lower fertility rates and economic progress.

Dr. Sahlu Haile, African Advisor for the David and Lucile Packard Foundation, had worked on family planning programming in Kinshasa in the 1980s. He returned in 2009 for an exploratory visit and again in June 2012 to speak at the meeting on Advocacy for the Financing of Family Planning in the DRC. During his presentation at the conference and in 3 subsequent visits to the country, he was able to draw parallels between the situation in Ethiopia (in the years before family planning had yet to take off) and the DRC (currently at a similar point in its history). His return visits to the DRC and relentless encouragement to implementing partners-despite the obvious challenges-allowed government officials and implementing partners to believe that increasing contraceptive prevalence at the national level was in fact an attainable objective.

### Increased Technical and Financial Support From Donors

Historically, USAID and UNFPA have been the primary donors for family planning in the DRC. Between 2002 and 2014, USAID’s budget for family planning in the DRC increased steadily. The World Bank also funded family planning as part of integrated health programming through June 2014 through the *Project d’Appui à la Réhabilitation du Secteur de la Santé* (PARSS) (Health Sector Support Project); similarly, DFID has supported family planning through its rural health-strengthening project, *Projet d'Accès aux Soins de Santé Primaire* (Access to Primary Health Care Project).

Since 2012, new donors have entered the DRC. The Bill and Melinda Gates Foundation supported a project to build the evidence base for family planning in Kinshasa, starting in 2010, and then expanded its activities to include advocacy work (through the Advance Family Planning initiative of the Johns Hopkins Bloomberg School of Public Health, which supported work in preparation for the Addis declaration) and improved monitoring and evaluation of contraceptive uptake (through the projects PMA2020 [Performance Monitoring and Accountability 2020] and Track 20). In 2014, the Gates Foundation provided additional support for strengthening the contraceptive logistics system, introducing the subcutaneous injectable Sayana Press, and piloting a system to track contraceptive stock levels using cell phones.

Other new donors in the DRC include the Packard Foundation, which funded a major initiative to increase access to, quality of, and demand for family planning services in Kinshasa. In addition, Agence Française de Développement (AFD) (French Development Agency) began supporting the demographic analysis of the consequences of population growth, as well as collaborating with the PARSS project of the World Bank. In 2013, the Norwegian government allocated US$20 million to a population and environment project. Furthermore, while USAID funding has continued at a fixed level for population activities, the national family planning program has benefitted from additional funding from PEPFAR (the United States President’s Emergency Plan for AIDS Relief). The identification of family planning as the second pillar of preventing mother-to-child transmission of HIV has resulted in the introduction of new family planning services in Kinshasa and elsewhere in the country.

Signs of increased government support of family planning and the existence of a costed strategic plan provide donors with additional justification to invest in the national program, creating a virtual cycle. Several other organizations are currently exploring their entry into family planning activity in the DRC, and recently the European Union committed 150 million euros to health programs, which include those to address MDG 4 and 5, beginning in 2014.^16^

Increased government support of family planning has encouraged increased donor support.

## POSSIBLE THREATS TO DURABILITY OF THE MOMENTUM

Given how recently this change in the family planning policy environment has occurred (basically, since 2012), the question arises whether it will have staying power or whether it will be reversed as quickly as it came about.

The most positive argument for sustained government commitment is that key government authorities and divisions have been involved in developing, validating, and approving important documents that signal this engagement (e.g., the General Secretary for Health, the DEP, PNSR, PNSA, D10, and others). Moreover, the current alignment of vision between the government, local implementing partners, and external donors serves to reinforce this commitment. Certain groups within civil society (e.g., *Cadre Permanent de Concertation des Femmes Congolaises* [CAFCO], or the Permanent Consultative Framework of Congolese Women) currently play a key role in advocating laws in favor of reproductive health and sustaining government commitment to family planning (Box 1).

However, continued government commitment is by no means guaranteed; multiple threats exist. First, the periodic reshuffling of DRC government officials may lead to the departure of key personnel who have been instrumental in championing family planning in the DRC. Second, although the government allocated funds for the procurement of contraceptives and supplies in 2013, there is no fixed line item for this activity in the MOH budget, risking possible reduction or loss of this support if there are competing priorities. Third, the country is expected to enter the electoral period when the current presidential term ends in 2016. The election process could trigger political instability and uncertainty, which could cause external donors to reduce or suspend their aid to the DRC. Finally, in 2013 the country made large strides in consolidating peace in the east. However, the challenge ahead is demobilizing and reintegrating combatants from eastern Congo into mainstream society. If the country fails to secure peace, the fragile progress made in family planning (and many other sectors) may be jeopardized by another cycle of violence.

Potential threats to sustained government commitment are many, including turnover in government staffing and lack of a fixed line item for family planning in the MOH budget.

## DISCUSSION

For long-time observers of the policy environment for family planning, the recent changes in the DRC are unprecedented and groundbreaking. At the high levels of government, family planning is no longer a neglected area for intervention but rather an activity that has gained visibility and political support. Key to spurring this change was the expressed support from Office of the Prime Minister and the directives for the relevant Ministries to take explicit action to prioritize family planning (Box 3).

**BOX 3. Key Ingredients to Family Planning Policy Change in the DRC**

- Directives from the highest levels of government (i.e., Office of the Prime Minister) effectively increased involvement and concrete actions on the parts of several Ministries (e.g., Planning, Health, and Gender).
- Developing a cohesive group of family planning stakeholders—from government, implementing agencies, and donors—took time but proved to be an invaluable investment (e.g., CTMP, Kinshasa Family Planning Coalition).
- Concrete achievements—such as the DRC government's Statement of Commitment in Addis Ababa, the launch of the Strategic Plan for Family Planning, granting official status to the CTMP, and a growing body of family planning research—have contributed to the momentum and have increased morale for those working in family planning.

A national law addressing reproductive health and family planning in the country is currently making its way through a series of judicial and legislative reviews, providing further evidence of the momentum for family planning (Box 1). This favorable reproductive health and family planning law includes comprehensive measures such as the protection of legal ethics and quality of life; improvement of gender relations; accountability of public authorities, civil society, and local communities; fertility assistance; availability of contraceptives and quality services; and the decriminalization of reproductive health. Whereas some might consider the multiple steps outlined in Box 1 as evidence of stalling, others familiar with the situation believe the multiple steps are a good-faith effort to ensure that all possible considerations are reviewed before the proposition comes up for a vote in the National Assembly.

Although much has been achieved at the central level, this progress has yet to diffuse to the 10 other provinces of the DRC, which is a vast country equivalent to the size of the United States east of the Mississippi River or of Western Europe. The Provincial Ministers of Health and Provincial Medical Directors have participated in key events relating to the acceleration of the MDGs (including family planning) and the development of the Strategic Plan for Family Planning in the DRC: 2014–2020. However, much policy work remains to be accomplished in the provincial capitals, with the aim of translating policy to action (e.g., family planning service delivery and demand generation) at the local level. In fact, because of the prolonged conflict in the eastern Congo, there has been a proliferation of NGOs in Kivu Province that work in reproductive health, gender-based violence, and family planning. Although we have no data to demonstrate cause-and-effect, North Kivu had the third highest modern contraceptive prevalence rate (mCPR) in the DRC as of 2013.^5^

Much work remains to be done at the local level to translate national policy to programmatic action.

Similar changes in the family planning policy environment have begun to occur in other francophone African countries, which generally have made less progress in family planning than their anglophone counterparts.^17^ For example, Senegal’s policy environment for family planning has shifted to include more concrete and explicit support from leaders in recent years. In 2011, the government hosted the International Conference on Family Planning, and in 2012 it made a financial commitment to double the national budget for contraceptive procurement the following year. The national program has tested and/or adopted innovative programming supported by research, including use of the “informed push model” for contraceptive logistics management and a pilot program to introduce Sayana Press.^18^ Although there are likely many contributing factors, modern contraceptive prevalence jumped from 12% to 16% between 2011 and 2013.^19^

Similar positive changes in the family planning policy environment have begun to occur in other francophone African countries.

Additionally, 8 francophone countries took part in a regional meeting in 2011 entitled “Population, Development, and Family Planning in West Africa: An Urgency for Action.” Also known as the Ouagadougou Partnership, the multi-agency delegations developed action plans for strengthening family planning programs and policies in their countries. Subsequently, several of these countries have made additional progress in this realm.^20^ For instance, in 2011, Burkina Faso committed a line item in its national budget for contraceptive procurement.

The policy environment is also improving in Côte d’Ivoire. Prior to 1991, the government actively discouraged family planning services. The IPPF member association, the Association Ivorienne Pour le Bien-Être Familial (AIBEF), was prohibited from publicizing its services, and provision of contraceptive methods to the public sector was restricted. Since then, the government position on family planning has improved, but use of contraception remains low. However, as of 2011, the government made more concrete commitments to improving contraceptive prevalence when President Alassane Ouattara issued a declaration on maternal health, including increasing family planning availability via health facilities from 60% in 2010 to 100% by 2015; expanding method access for women living with HIV and for youth; and increasing contraceptive commodities by including them in the recommended list of essential medicines to be subsidized and made more affordable.^21^

Niger, the fastest growing country in the world, was characteristic of the countries of francophone Africa that historically gave little priority to family planning. However, in 2013 political commitment changed dramatically with a quadrupling of its family planning budget for that year. Also, the government has taken several innovative actions, such as authorizing community health workers to provide Sayana Press, creating new mobile clinic services for isolated communities, and integrating family planning in the school health curriculum.^22^

Niger, the fastest growing country, quadrupled its family planning budget in 2013.

The larger question is whether change in the policy environment of a country will lead to effective programmatic action that translates into increased contraceptive use and decreased fertility rates. One marker of political support is an official population policy, but literature on its effect is mixed. Some of the most effective programs in the world have been led by countries with strong official population policies (e.g., China, Indonesia, and Mexico, to name a few).^23^ In other countries, especially in Latin America, NGOs provided leadership in family planning in the early years when governments still feared political fall-out, especially from the Catholic Church, for endorsing family planning.^24^ Conversely, not all countries that promulgated a strong population policy converted this into increased contraceptive use (e.g., Ghana, which in 1969 had one of the first population policies in sub-Saharan Africa but did not see much increase in its mCPR until 3 decades later).^25^ In earlier years, population policy often signaled that family planning programming was well underway rather than a driver of the process.^26^ In the context of francophone sub-Saharan African countries, government commitment would seem to be a prerequisite to meaningful change in family planning programming and contraceptive use. However, verbal support for policies and programs is insufficient to trigger meaningful change; it must be accompanied by financial support and programmatic action.

Observers of the international family planning movement will follow the promising policy advances in the DRC with great interest. Given the significant cultural and financial barriers to family planning in the DRC, change—to the extent it occurs—will be gradual. The recent advances in the policy environment represent an unprecedented platform on which to work toward this change.

**Figure 1:**
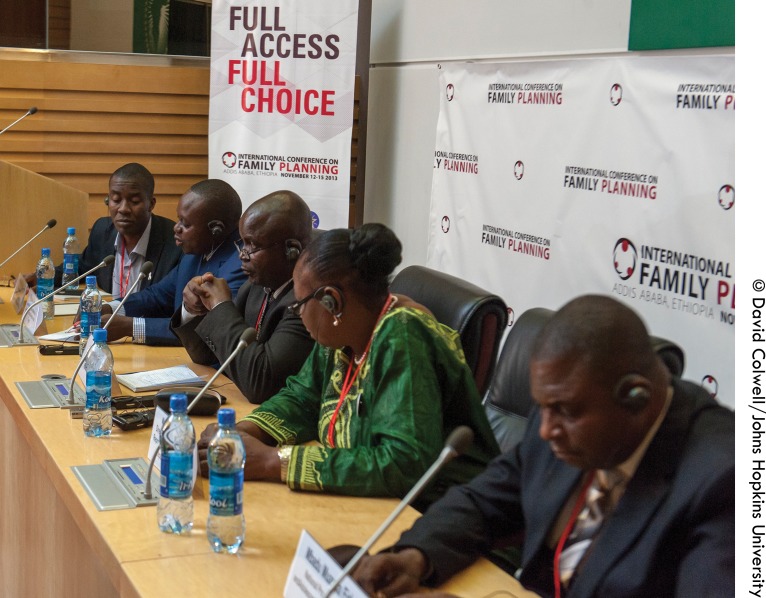
In November 2013, DRC government and NGO representatives held a press conference at the Third International Conference on Family Planning in Addis Ababa, where the government presented its Declaration of Commitment to Family Planning. Left to right: Dr. Arsene Binanga (Tulane’s Director of Family Planning Programs in the DRC), Dr. Thomas Kataba (of the Ministry of Health’s Studies and Planning Directorate), Mr. Dieudonné Kwete (advisor to the Prime Minister), Dr. Therese Kyungu (Director of the National Program of Reproductive Health), and Mr. Mbadu Mwanda (Director of the National Program for Adolescent Health).

## Notes

**Peer Reviewed**
